# Prioritizing biological pathways by recognizing context in time-series gene expression data

**DOI:** 10.1186/s12859-016-1335-8

**Published:** 2016-12-23

**Authors:** Jusang Lee, Kyuri Jo, Sunwon Lee, Jaewoo Kang, Sun Kim

**Affiliations:** 1 Department of Computer Science and Engineering, Seoul National University, Seoul, Republic of Korea; 2 Department of Computer Science and Engineering, Korea University, Seoul, Republic of Korea; 3 Interdisciplinary Program in Bioinformatics, Seoul National University, Seoul, Republic of Korea; 4 Bioinformatics Institute, Seoul National University, Seoul, Republic of Korea

**Keywords:** Literature information, Pathway, Pathway analysis, Pathway prioritization, Time series

## Abstract

**Background:**

The primary goal of pathway analysis using transcriptome data is to find significantly perturbed pathways. However, pathway analysis is not always successful in identifying pathways that are truly relevant to the context under study. A major reason for this difficulty is that a single gene is involved in multiple pathways. In the KEGG pathway database, there are 146 genes, each of which is involved in more than 20 pathways. Thus activation of even a single gene will result in activation of many pathways. This complex relationship often makes the pathway analysis very difficult. While we need much more powerful pathway analysis methods, a readily available alternative way is to incorporate the literature information.

**Results:**

In this study, we propose a novel approach for prioritizing pathways by combining results from both pathway analysis tools and literature information. The basic idea is as follows. Whenever there are enough articles that provide evidence on which pathways are relevant to the context, we can be assured that the pathways are indeed related to the context, which is termed as relevance in this paper. However, if there are few or no articles reported, then we should rely on the results from the pathway analysis tools, which is termed as significance in this paper. We realized this concept as an algorithm by introducing Context Score and Impact Score and then combining the two into a single score. Our method ranked truly relevant pathways significantly higher than existing pathway analysis tools in experiments with two data sets.

**Conclusions:**

Our novel framework was implemented as ContextTRAP by utilizing two existing tools, TRAP and BEST. ContextTRAP will be a useful tool for the pathway based analysis of gene expression data since the user can specify the context of the biological experiment in a set of keywords. The web version of ContextTRAP is available at http://biohealth.snu.ac.kr/software/contextTRAP.

**Electronic supplementary material:**

The online version of this article (doi:10.1186/s12859-016-1335-8) contains supplementary material, which is available to authorized users.

## Background

The advancement of gene profiling techniques has expanded the genomics research from a single gene analysis to the analysis of genome-wide gene expression data [1, 2]. The result from genome-wide gene expression data analysis is typically further processed for pathway analysis to investigate the association between a set of genes or proteins and phenotypes such as metabolism [3], gene regulation [4] or signal transduction [5]. Pathway analysis produces the global landscape of cellular process [6], which cannot be derived from a list of differentially expressed genes (DEGs). Especially, understanding the dynamics of pathways helps identify biological processes triggered by a specific condition [7, 8] or elucidate a different mechanism among multiple phenotypes [9, 10].

A lot of efforts have been made to define sets of genes that perform key roles for common mechanisms. As a result, a number of databases have been developed to curate sets of genes as pathways [11]. KEGG is the most widely used pathway database and it also provides graphical representations for molecular interactions in pathway [12]. REACTOME [13] and NCI-PID [14] are also well curated pathway databases used for many research projects. Pathway databases facilitate gene set analysis and help researchers to understand biological process.

With gene expression profiling techniques and well curated pathway databases, gene expression data is now routinely analyzed in terms of biological pathways. Over the years, a number of tools for pathway analysis have been developed and they can be categorized as i) over-representation analysis (ORA), ii) functional class scoring (FCS), and iii) pathway topology (PT) based approach [15]. ORA methods select a gene set (e.g. DEGs) from expression data and statistically evaluate the proportion of the gene set in terms of biological pathways. Fisher’s exact test or Chi-square are widely used to perform the ORA based analysis tasks [16]. FCS methods assign gene-level statistics to each gene, and aggregate them into the pathway-level statistics. Gene set enrichment analysis (GSEA) is a representative FCS method that determines whether a set of genes that are predicted to share a common biological function are randomly distributed or over-represented either at the top or bottom of the ranked list [17]. PT-based methods use the topology of a pathway where genes are nodes and their interactions are edges. For instance, CliPPER selects significant pathways based on the network represented as the mean and covariance matrix and determines fraction of signaling paths that are correlated with phenotypes [18].

Recently, time-series data has been considered as important key resources to understand the dynamics of biological mechanism over time and the number of datasets or research projects producing time-series gene expression data has increased dramatically [19]. Thus, several pathway analysis methods for time-series gene expression data have also been developed recently. For example, Time-series RNA-seq Analysis Package (TRAP) analyzes time-series gene expression data and identifies significant pathways with regard to the propagation difference of gene expression between two different conditions [20].

## Motivation

Pathway analysis from gene expression data using these tools identifies which biological pathways are important to understand the context of data or research being investigated (e.g. phenotype). However, there is no guarantee that all pathways selected by pathway analysis are relevant to the context [21]. One of the major reasons for this inconsistency is existence of overlapped genes among multiple pathways [22, 23]. Table 1 shows how many genes belong to multiple pathways in KEGG pathway database. Among 6,972 genes participating in 295 homo sapiens pathways of KEGG, more than half of the genes belong to more than two pathways. As an example, a single gene, *MAPK1*, is involved in 85 pathways. These overlapped genes among multiple pathways make some pathways significant regardless of the relation with the context, concurrently. Thus, the result of pathway analysis can produce pathways that are not related to the context being investigated.

**Table 1:** The number of involved pathways for each gene in KEGG pathway database

The number of involved pathways	The number of genes
1	3157
2∼10	3405
11∼20	264
21∼30	70
31+	76
The number of total pathways	The number of total genes
295	6972

It shows how many genes belong to multiple pathways of homo sapiens in KEGG database. Among 6,972 genes that consist of 295 pathways, more than half of the genes belong to two or more pathways

One effective way to verify whether a significant pathway is actually related to the context or not is to search the literature information. If some literatures support specific relationship between the pathway and the context of data, we can be more confident to choose the pathway as one significantly expressed and truly related to the context. Thus, our goal in this study is to come up with a computational framework to combine pathway analysis of gene expression data and the literature information to select pathways relevant to the the context of the experimental condition, typically control vs. treated. To describe our research clearly throughout the paper, we introduce two concepts as below.

- **Significance**: It is to measure the correlation between a pathway and the context from *gene expression data*[24]. Currently existing pathway analysis tools evaluate which pathways are significant in characterizing phenotype, using it in terms of scores like p-values. In this paper, this concept is defined as *significance* and it will be used throughout the manuscript.
- **Relevance**: It means how a pathway is truly associated to the context [25] and one practical method to measure it is to exploit *literature information* reporting the observations or evidences of association between the pathway and the context. It is stronger than *significance* since *significance* is simply to measure how much correlation exists between the pathway and the context through expression values, while *relevance* requires direct observations or evidences that the pathway is actually related to the context. In this paper, this concept is defined as *relevance* and it will be used throughout the manuscript.

**Fig. 1:**
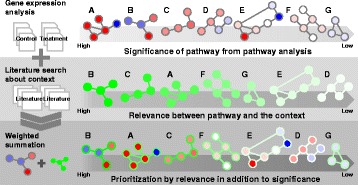
The overview of our proposed method. The *significance* of pathways are obtained from pathway analysis using gene expression data. The *relevance* of pathways are retrieved from literature information. Then, *significance* and *relevance* are integrated into a single score in a weighted sum. The integrated score is used to prioritize pathways, considering *significance* and *relevance*, simultaneously

The goal of this study is to come up with a computational method to combine both *significance* and *relevance*. In particular, the integration of these two concepts is to combine analysis of gene expression data (*significance*) and the literature information based on the contextual information provided by the user (*relevance*). Figure 1 shows the overview of the proposed method. The *significance* of pathways is calculated using existing pathway-based gene expression data analysis tools. The *relevance* of pathways is obtained from the literature search upon keywords that are provided by the user to specify the context of the experiment. Scores for *significance* and *relevance* are combined into a single score by summing up the two scores as a weighted sum.

## Methods

For the implementation of the proposed concept, we used an existing pathway analysis method and a literature search tool. TRAP [20] is selected for pathway analysis. TRAP uses method that combines the ORA and PT-based approaches to find significant pathways from KEGG pathway database and it is also designed for time-series gene expression data. For the literature search, Biomedical Entity Search Tool (BEST) [26] is used. BEST uses the concept of Maximal Coherent Semantic Unit for indexing keywords to associate the keyword and literatures efficiently. Using BEST, users can specify the contextual information by specifying a set of keywords for the biological experiments that generated data for analysis. BEST returns biological entities with entity scores as a result of literature search. The entity score is computed by considering various factors such as the publication date, the number of citations and impact factors of journals.

**Fig. 2:**
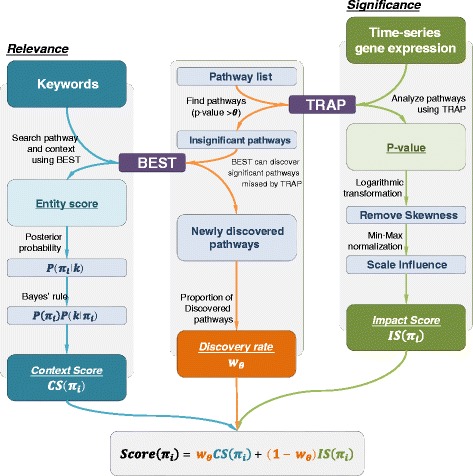
The workflow of ContextTRAP. Time-series gene expression data is given to TRAP and keywords representing the context are given to BEST as input. ContextTRAP incorporates TRAP and BEST by integrating the analysis results from both tools. The entity score derived from BEST with keywords is converted to *Context Score (CS)*, which measures the *relevance* of pathway. For that, the concept of posterior probability and Bayes’ rule are used. The *p*-value, the result of TRAP, is transformed and normalized to *Impact Score (IS)* measuring the *significance* of pathway. Then, *Discovery rate* is automatically determined as a weight for integration of two scores, depending on the results of TRAP and BEST. Finally, the integrated score is used to prioritize pathways in terms of both *significance* and *relevance*

The integration of *significance* and *relevance* can be easily done with these two tools. Integrating TRAP and BEST, we implemented ContextTRAP as in Fig. 2. Time-series gene expression data and keywords representing a context of data are given as inputs. TRAP analyzes time-series gene expression data to obtain the *significance* of pathways. Using keywords, BEST is used to obtain the *relevance* between a pathway and the context as an entity score.

To quantify and integrate *significance* and *relevance*, we introduce two score terms. Using the concept of posterior probability, *Context Score (CS)* measures the *relevance* of pathway from the entity score of BEST. Meanwhile, *Impact Score (IS)* measures the *significance* of pathway from *p*-value result of TRAP analysis for time-series gene expression data. Then, *CS* and *IS* are integrated via an automatically assigned weight called *Discovery rate*.

In this section, we introduce how to transform the result of BEST to *CS*, how to transform the result of TRAP to *IS*, and how to calculate *Discovery rate* that will be used as weight to integrate of *CS* and *IS*. In addition, we introduce the method of Pathway Set Enrichment Analysis (PSEA) to evaluate the result of ContextTRAP in Section “Accuracy of discovery rate estimation”.

### Context score

The set of pathways to be analyzed is denoted as *π*={*π*_1_,…,*π*_*n*_}. *π* has *n* pathways and *i*-th pathway is denoted as *π*_*i*_. A keyword is needed to be specified as an input for BEST which is the contextual information related to the context like the experimental condition or the phenotype which the research wants to investigate (e.g. disease, symptom, or gene). This keyword is denoted as *k*.

*Context Score (* *CS*(*π*_*i*_,*k*)*)* measures how many research or articles reported some association of the pathway *π*_*i*_ and the keyword *k*. *C* *S*(*π*_*i*_,*k*) can be computed as a form of posterior probability like Eq. (1). By entering *k* into BEST as a search word, a list of entities related to *k* is retrieved with an entity score of each entity. Then, *C* *S*(*π*_*i*_,*k*) is calculated easily from the entity score of *π*_*i*_ by finding *π*_*i*_ from the retrieved list of entities.

1 $$ CS(\pi_{i}, k) = P(\pi_{i}|k)  $$

However, some pathways are not included as entities in BEST, probably because the current literatures, though increasing rapidly, are yet to be complete enough to cover all biological pathways. Thus, it is difficult to get the entity score for whole pathways by Eq. (1). To handle this problem, we converted *P*(*π*_*i*_|*k*) to Eq. (2) using the Bayes’ rule. Then, we compute likelihood *P*(*k*|*π*_*i*_) instead of *P*(*π*_*i*_|*k*), meaning that retrieves the entity score of *k* entering *π*_*i*_ as a search word into BEST. Considering this concept, if user select *k* from the entity list of BEST in advance, *C* *S*(*π*_*i*_,*k*) of any pathway can be obtained from the entity score of *k*.

2 $$ P\left(\pi_{i}|k\right) = \frac{P\left(\pi_{i}\right)P\left(k|\pi_{i}\right)}{P(k)} =\frac{P\left(\pi_{i}\right)P\left(k|\pi_{i}\right)}{\sum_{j=1}^{n}{P\left(\pi_{j}\right)P\left(k|\pi_{j}\right)}}   $$

Given a pathway *π*_*i*_ that is used as a search word for BEST, the entity score of *k* is transformed to *P*(*k*|*π*_*i*_) as Eq. (3). It is a logarithm of (*s*_*i*_+1) to base *b*. *s*_*i*_ is the entity score of *k* derived from using *π*_*i*_ as search word and *b* is *s*_*max*_+2, where *s*_*max*_ is the maximum of all *s*_*i*_. Logarithm is a monotonically increasing function, so a higher entity score is mapped to a higher probability. Because the base of logarithm is *s*_*max*_+2, the highest entity score is mapped to a probability approximated to 1. Thus, Eq. (3) converts entity scores of *k* for each of pathways ranged to [0,*s*_*max*_] to a probability with a range [0,1). In addition, this function is concave down, which means the rate of increase is larger for smaller entity scores but it decreases and converges gradually. Thus, use of the logarithm function effectively makes densely distributed small entity scores widely scattered while big entity scores remain distinguishable from the small entity scores.

3 $$ P\left(k|\pi_{i}\right) = \log_{b}{\left(s_{i} + 1\right)}   $$

4 $$ P\left(\pi_{i}\right) = \log_{b}{\left(t_{i} + 1\right)}   $$

Prior probability *P*(*π*_*i*_) is calculated using Eq. (4), which is similar to Eq. (3). *P*(*π*_*i*_) represents the prevalence of *π*_*i*_ in all possible keywords, and this prevalence can be acquired by *t*_*i*_ derived from summing up all scores of entities related to *π*_*i*_ in BEST. Because obtaining scores of all possible entities is a time-consuming work and scores of low-ranked entities have very small values, *t*_*i*_ is approximated by entering *π*_*i*_ into BEST and adding top ten entity scores in three categories (gene, disease, and pathway), respectively. Then, *b* is calculated as *t*_*max*_+2 to make *P*(*π*_*i*_) range from 0 to 1, where *t*_*max*_ is the maximum of all *t*_*i*_.

In some cases, multiple keywords are needed to cover the context of data. Then, final *C* *S*(*π*_*i*_,*k*) of multiple keywords is obtained by calculating the average of *C* *S*(*π*_*i*_,*k*) derived by each keyword.

### Impact score

*Impact Score (* *IS*(*π*_*i*_)*)* measures how significant pathway *π*_*i*_ is as a result of gene expression data analysis. To handle time-series data, ContextTRAP uses TRAP for analyzing gene expression data. Before deriving *I* *S*(*π*_*i*_), we define *I* *S* ^∗^(*π*_*i*_) like Eq. (5), using the p-value of each pathway from the result of TRAP. Because the p-values of significant pathways are very small near to zero, distribution of *p*-values has to be scattered and expanded. Thus, a negative logarithm to base 10 is applied for negating the density of p-values.

5 $$ IS^{*}(\pi_{i}) = -\log_{10}{pvalue_{i}}  $$

After applying the logarithm-based transformation, a min-max normalization adjusts the maximum (or minimum) of *I* *S* ^∗^(*π*_*i*_) to the maximum (or minimum) of *C* *S*(*π*_*i*_,*k*) like Eq. (6). Then, *I* *S*(*π*_*i*_) can have an equal influence as *C* *S*(*π*_*i*_,*k*) when integrated into a single combined score.

6 $$ {}\begin{aligned} IS(\pi_{i})\! = \left(IS^{*}(\pi_{i})-\min{IS^{*}}\right)\frac{\max{CS}-\min{CS}}{\max{IS^{*}}-\min{IS^{*}}}+\min{CS} \end{aligned}  $$

### Discovery rate

*IS*(*π*_*i*_) and *CS*(*π*_*i*_,*k*) are normalized in the same scale so that the integration of the two scores can be easily done. Two scores are integrated by a weighted sum as in Eq. (8), so it is important to select a proper weight for integration reflecting the importance of *IS*(*π*_*i*_) and *C* *S*(*π*_*i*_,*k*). *Discovery rate* (*w*_*θ*_ *)* is a dynamically assigned weight as in Eq. (7) to reflect the importance of the *significance* and the *relevance*. It is the proportion of pathways whose p-value is bigger than a specific threshold *θ* and *C* *S*(*π*_*i*_,*k*) is bigger than zero. It represents the ratio of pathways that are not selected as significant by TRAP but discovered as meaningful in BEST. In other words, *Discovery rate* shows how many pathways are missed by the gene expression analysis but have been investigated and reported in the literature v.s. all the pathways. *n* is the total number of pathways and *θ* means a threshold of p-value for selecting insignificant pathways from TRAP and 0.05 is used in this paper, since p-value of 0.05 is a widely used cutoff value for indicating statistical significance. *Discovery rate* reflects some characteristics about data. 1) If the user-defined context is well supported by the literature and BEST can cover many pathways related to the context, *Discovery rate* will be bigger to increase the effect of *C* *S*(*π*_*i*_,*k*) and vice versa. 2) Although BEST finds many pathways with the context, if TRAP covers most of the pathways detected by BEST, the importance of *C* *S*(*π*_*i*_,*k*) decreases.

7 $$ w_{\theta} = \frac{\sum_{i=1}^{n}{I(pvalue_{i} > \theta \ and \ CS(\pi_{i}, k) > 0)}}{n}  $$

The final score of *π*_*i*_ is derived by combining *C* *S*(*π*_*i*_,*k*) and *I* *S*(*π*_*i*_) with *Discovery rate* as in Eq. (8). Using this score, significance of pathway is re-estimated.

8 $$ Score(\pi_{i}, k) = w_{\theta}CS(\pi_{i}, k) + (1-w_{\theta})IS(\pi_{i})  $$

### Pathway set enrichment analysis

To evaluate the pathway list determined by ContextTRAP in Section “Accuracy of discovery rate estimation”, we propose Pathway Set Enrichment Analysis (PSEA), a modified version of Gene Set Enrichment Analysis (GSEA)[17] at the pathway level. GSEA is a method to determine whether a set of genes is significant or not, while PSEA measures whether a ranked list of pathways is significant or not. From a pathway list ranked by Eq. (8), PSEA calculates Rank Score (*RS*(*i*)) at rank *i* like Eq. (9). In the ranked list, *R* *S*(*i*) is increased or decreased from *R* *S*(*i*−1), depending on whether *π*_(*i*)_ is relevant pathway or not, where *π*_(*i*)_ is i-th pathway in the ranked list. In this paper, relevant pathways are defined as *π* ^∗^ by DAVID [27, 28] analysis using a specific gene set that was validated or reported to be related to the context in original paper, while the rest of the pathways are defined as *π* ^−^. Firstly, *R* *S*(0) is initialized to 0. Then, if *π*_(*i*)_ is involved in *π* ^∗^, *R* *S*(*i*) is increased by a proportion of *r*_*i*_ which means the impact of rank *i*, where *r*_*i*_ is *n*−*i*+1. On the other hand, if *π*_(*i*)_ is member of *π* ^−^, *R* *S*(*i*) is decreased by a reciprocal of the number of *π* ^−^. Enrichment Score (*ES*) is the maximum of *RS*, which measures how significant the pathway list is. The higher the ranks of overall *π* ^∗^, the higher the *ES* score is.

9 $$ RS(i)= \left\{ \begin{array}{ll} 0, & i = 0 \\ RS(i-1) + \frac{r_{i}}{\sum{}^{}{r}},& \pi_{(i)} \in \pi^{*} \\ RS(i-1) - \frac{1}{\left|\pi^{-}\right|}, & \pi_{(i)} \in \pi^{-} \end{array}\right.  $$

10 $$ ES = \max_{i}{RS(i)}  $$

## Results and discussion

In this section, we evaluated the performance of ContextTRAP by analyzing two public datasets. Two datasets have time-series gene expression data measured in control vs. treatment experiments. Using these datasets, we evaluated the performance of ContextTRAP in various ways. Firstly, we tested whether the literature information truly supports the *relevance*, using various keywords, each of which has different strength of representing the context of data. Secondly, Pathway Set Enrichment Analysis (PSEA) introduced in Section “Pathway set enrichment analysis” was used to verify whether *Discovery rate* estimates a proper weight or not for integration. Next, Kolmogorov Smirnov (KS) test was used to compare the performances of ContextTRAP and the original TRAP in terms of prioritization. Finally, we compared ContextTRAP with original TRAP and also with three existing pathway analysis methods, one from each of major pathway analysis categories of ORA, FCS and PT-based methods, in terms of F_1_ score. Additionally, we investigated the biological importance of the prioritized pathways in ContextTRAP.

### Data processing

To evaluate the performance of ContextTRAP, two public datasets are selected. i) *H5N1* identified signaling networks affected by highly pathogenic avian influenza H5N1 infection [29]. ii) *Th17* identified regulatory networks controlling the *T*_*H*_17 cell differentiation triggered by TGF- *β*1 and IL-6 [30]. Raw data of two datasets have been deposited in the Gene Expression Omnibus(GEO) database under access links http://www.ncbi.nlm.nih.gov/geo/query/acc.cgi?acc=GSE28166 for *H5N1* and http://www.ncbi.nlm.nih.gov/geo/query/acc.cgi?acc=GSE43955 for *Th17*.

Firstly, keywords representing the context of data are necessary to obtain *CS*. It should be one of the entities of BEST, thus we selected the simplest and the most relevant keywords from the entity list in BEST. In Table 2, keywords used for each dataset are shown. *Influenzas* is used as a keyword for *H5N1*, while *TGFB1* and *IL6* are used as keywords for *Th17*. In the web version of ContextTRAP, user can search and select keywords from the entities of BEST to specify the experimental condition of the input dataset.

**Table 2:** Datasets used to evaluate contextTRAP

Dataset	Species	Keywords	Discovery rate	|*π*|	|*π* ^∗^|
*H5N1*	Homo sapiens	Influenzas	0.30	295	15
*Th17*	Mus musculus	TGFB1 and IL6	0.21	291	29

*H5N1* is a time-series gene expression data of homo sapiens and about highly pathogenic avian influenza H5N1 infection. *Influenzas* was used as keyword and *Discovery rate* was calculated as 0.30. *Th17* is a time-series gene expression data of mus musculus and about *T*_*H*_17 cell differentiation. *TGFB1* and *IL6* were used as keywords and *Discovery rate* was calculated as 0.21. |*π*| represents the number of total pathways and |*π* ^∗^| is the number of the relevant pathways retrieved from DAVID with validated genes

To define the pathways which are relevant to the context of each dataset, DAVID [27, 28] analysis was performed using gene sets validated and reported in experiments that generated each dataset. The gene sets and the selection criteria of the gene sets are provided in additional file [see Additional file 1]. With these gene sets, 15 pathways from *H5N1* and 29 pathways from *Th17* are selected as relevant pathways of each dataset. In this paper, these relevant pathways are represented as *π* ^∗^ and the rests are represented as *π* ^−^. Then, the purpose of our research is prioritizing *π* ^∗^ from pathway analysis result. The list of *π* ^∗^ for each dataset is provided in additional file [see Additional file 2].

For running TRAP algorithm, we selected time-lag factor, parameter to adjust the ratio of influence from the previous time point, as 1. Also, cutoff value, threshold to find DEGs by fold-change as logarithm, was selected as 1, according to the default values.

Using the result of BEST and TRAP, *Discovery rate* was calculated automatically: 0.30 for *H5N1* and 0.21 for *Th17*. The result of data processing is shown in Table 2. *H5N1* is time-series gene expression data of *Homo sapiens* and has 295 KEGG pathways in total referred as |*π*|. *Th17* is for *Mus musculus* and has 291 KEGG pathways. The |*π* ^∗^| means the number of *π* ^∗^ which are context-relevant pathways retrieved from DAVID.

### The effect of relevance between keyword and the context of data

**Fig. 3:**
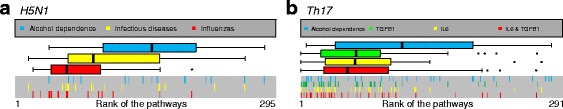
The comparative result of literature search from BEST with various keywords. **a** is the result of *H5N1*. *Alcohol dependence*, *Infectious diseases*, *Influenzas* are selected as keywords depending on the relevance to the context. *Boxplot* of each color shows the distribution of rank of relevant pathways (*π* ^∗^) in pathway list sorted by BEST score returned with each keyword and color marking in the *bottom gray* boxes is the position of *π* ^∗^ in pathway list. It shows relevant pathways rank higher when more relevant keyword is given to BEST. **b** is the result of *Th17*. *Alcohol dependence*, *TGFB1*, *IL6* and combination of *TGFB1* and *IL6* are used as keywords. It shows that combination of multiple relevant keywords can make better performance than using a single keyword

To show whether the literature information can represent the *relevance*, we tested whether the distribution of *π* ^∗^ in pathway list returned from BEST is different in accordance with the degree of association between keyword and context of data. Figure 3 shows pathway list sorted by the result returned from BEST with various keywords for each dataset.

Figure 3(a) is the result of *H5N1* using *Alcohol dependence*, *Infectious diseases* and *Influenzas* as keywords. *Influenzas* is the best keyword well representing the context of *H5N1* and *Infectious diseases* that is more comprehensive term than *Influenzas* was selected as secondly relevant keyword. *Alcohol dependence* was selected to show the result of keyword having little relevance with the context. In the bottom of the figure, each color marker shows the position of *π* ^∗^ in 295 pathways ordered by BEST score derived from each keyword. Boxplot above shows the distribution of rank of *π* ^∗^ in pathway list. In *Influenzas* which is most relevant to *H5N1*, *π* ^∗^ are mainly positioned at the front of pathway list. It means *Influenzas* well represents the context of *H5N1*. As a secondly relevant keyword, *Infectious diseases* shows similar result with *Influenzas*, but *π* ^∗^ are mainly positioned at lower rank than *Influenzas*. In *Alcohol dependence*, most *π* ^∗^ are distributed in backward of pathway list. It suggests that the result of literature search with a keyword that well reflects the context of data makes reasonable pathway scores representing the *relevance* between pathway and the context.

Figure 3 b shows the result of *Th17* using *Alcohol dependence*, *TGFB1*, *IL6* and combination of *TGFB1* and *IL6* as keywords. Like Fig. 3 a, 291 pathways ordered by the result of BEST with various keywords are represented in x-axis and *π* ^∗^ are marked in color. The result of *Alcohol dependence* which is a irrelevant keyword shows that *π* ^∗^ are uniformly distributed on the pathway list without any tendency. Two mainly relevant keywords, *TGFB1* and *IL6*, show good performance where *π* ^∗^ are focused on top of list. When two relevant keywords are used together like a combination of *IL6* and *TGFB1*, however, the result is improved than the result of single keyword. It seems that combined keywords mutually supplement search results and make synergy from each keyword.

### Accuracy of discovery rate estimation

**Fig. 4:**
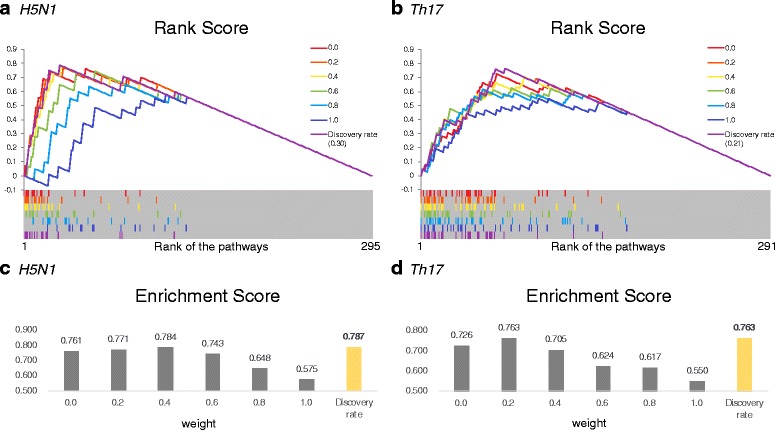
The result of pathway set enrichment analysis. **a** – **b** show PSEA results of *H5N1* and *Th17*, respectively. The pathway list sorted by *Score*(*π*_*i*_,*k*) with various weights, from 0.0 to 1.0 increased by 0.2 and *Discovery rate* (0.30 and 0.21), is present in x-axis. The *line graph* shows Rank Score (*R* *S*(*i*)) of each rank *i*, changed through pathway list from the first rank to the last rank. Color marking in the *bottom gray* boxes means position of *π* ^∗^ in pathway list. **c** – **d** show Enrichment Score(*ES*) of each dataset, which is the maximum *RS*

To show how well *Discovery rate*, the weight in Eq. (8), is set automatically, we used Pathway Set Enrichment Analysis (PSEA) introduced in Section “Pathway set enrichment analysis”. The weights increased by 0.2 from 0.0 to 1.0 were compared with *Discovery rate* of each dataset. A weight of 0.0 indicates that the TRAP analysis result is solely used and a weight of 1.0 indicates that the BEST analysis result is solely used. Figure 4 shows the results of PSEA for (a) *H5N1* and (b) *Th17*. In Fig. 4 a–b, the positions of *π* ^∗^ in the sorted pathway list determined by ContextTRAP are indicated in x-axis at 7 different weights, *π* ^−^ in gray and *π* ^∗^ as bars in color other than gray. The line graph shows a change of *R* *S*(*i*) through x-axis from the first rank to the last rank.

With the weight of 0.0 in Fig. 4 a–b, which shows the result of the original TRAP, the *π* ^∗^ generally tend to be focused on the front of pathway list, which indicates that TRAP performed well. However, considering the literature information, ContextTRAP prioritized *π* ^∗^ more from the result of TRAP. Figure 4 c–d shows *ES* which is the maximum *RS* at weights of 0.0, 0.2, 0.4, 0.6, 0.8, 1.0 and also *Discovery rate*. With *Discovery rate*, ContextTRAP improved *ES* compared to original TRAP, from 0.761 to 0.787 for *H5N1*, and from 0.726 to 0.763 for *Th17*. It is the highest of the results from each weight. This experiment shows that our strategy of combining *relevance* and *significance* of pathways is quite good without requiring the user to set the weight value. Note that our system determines *Discovery rate* automatically and it reduces the bias which can be caused by a fixed weight.

### How much improvement is achieved in detecting relevant pathways in comparison with the original version of TRAP

We measured how much improvement was achieved by comparing performance of ContextTRAP that incorporated BEST and the original version of TRAP that does not utilize literature information. Using Kolmogorov Smirnov (KS) test, we can compare two distributions effectively. KS test measures the *D* statistic that represents the maximum distance between two empirical distribution functions of two samples. Applying the KS test, we compared distributions of *π* ^∗^ and *π* ^−^ in sorted pathway list returned from ContextTRAP and original TRAP. If *π* ^∗^ are positioned at the head of list and *π* ^−^ are positioned at the tail of list, *D* between them becomes higher. If *π* ^∗^ and *π* ^−^ are mixed in list, on the other hand, *D* will be a lower value.

**Fig. 5:**
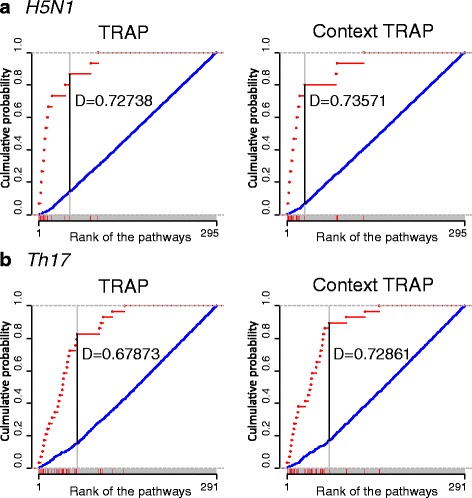
The result of KS test for the distribution of *π* ^∗^ and *π* ^−^ in pathway list returned from TRAP and ContextTRAP. **a** is the result from *H5N1* and **b** is the result from *Th17*. In the plot, *red* points indicate empirical distribution function of *π* ^∗^ and *blue* points indicate that of *π* ^−^. *Gray box* along the x-axis represents the pathway list retrieved from each method. Pathways are sorted by *p*-value (TRAP) and *S* *c* *o* *r* *e*(*π*_*i*_,*k*) (ContextTRAP). *Red markers* represent the positions of *π* ^∗^ in pathway list. *D* statistic from KS test represents distance between two empirical distribution functions and it shows improvement in ContextTRAP incorporating the literature information

Figure 5 shows the results of KS test of ContextTRAP and the original TRAP for each dataset. Figure 5 a is the result of *H5N1* and Fig. 5 b represents the result of *Th17*. The pathway list is sorted by score of each analysis and is present in x-axis of each plot. Red markers indicate the position of *π* ^∗^ in the pathway list. In each plot, points in red color represent the empirical distribution function of *π* ^∗^ and blue points indicate that of *π* ^−^ in the pathway list returned from pathway analysis.

For two datasets, plots show that *π* ^∗^ are distributed at the head of pathway list and empirical distribution function of *π* ^∗^ increases more rapidly in ContextTRAP compared with original TRAP. In addition, *D* is higher in ContextTRAP than original TRAP. It means *π* ^∗^ are prioritized well in ContextTRAP by using literature information.

### Comparison with other pathway analysis methods

We compared the performances of ContextTRAP with three other pathway analysis methods included in the graphite web server [31], also with the original TRAP. Graphite web is a web tool for pathway analysis using gene expression data, providing various analysis methods. For the comparison, three pathway analysis methods were selected, one for each of three categories of pathway analysis tools—Fisher’s exact test, GSEA [17] and CliPPER [18] represent ORA methods, FCS methods and PT-based methods, respectively. For a quantitative comparison, we calculated F_1_ score of a pathway list determined by each of the pathway analysis tools. F_1_ score is a harmonic mean of precision and recall and it is widely used to evaluate the performance of binary classification tests. In this analysis, we consider *π* ^∗^ set as a positive condition set and *π* ^−^ set as a negative condition set in terms of true condition set. Then, pathways are predicted as positive or negative, depending on whether a pathway is classified as significant or insignificant by each of pathway analysis tools. Using the result of predicted condition set and the pre-defined true condition set, F_1_ score is calculated.

To split the pathway list of ContextTRAP into significant and insignificant, p-value of each pathway is calculated by permutation. P-value is derived from a distribution of permuted scores by generating all possible combinations of *CS* and *IS* of all pathways. Then, pathways having p-value under 0.05 are selected as significant for ContextTRAP. For other pathway analysis tools, we selected pathways having p-value below 0.05 as significant.

**Fig. 6:**
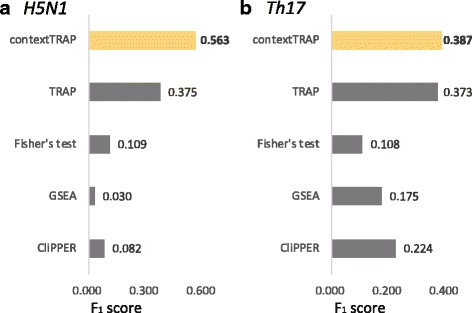
The F_1_ score of five pathway analysis methods for each dataset. **a** is the result of *H5N1* and **b** is the result of *Th17*. Fisher’s exact test, GSEA and CliPPER from graphite web were compared with ContextTRAP and also original TRAP was included in the comparison. ContextTRAP shows higher F_1_ score than other three methods and even than original TRAP

Figure 6 shows F_1_ scores for five methods—ContextTRAP, original TRAP, Fisher’s exact test, GSEA and CliPPER. Figure 6 a represents the result of *H5N1* and Fig. 6 b represents the result of *Th17*. For two datasets, original TRAP shows higher F_1_ score than other three methods—Fisher’s test, GSEA and CliPPER. It is probably because other methods except TRAP do not consider the effect of time factor in a proper manner, handling the time-series samples as multiple replicates. However, ContextTRAP shows the highest F_1_ score, even higher than the score of original TRAP. It indicates that literature search realizes the prioritization of context-relevant pathways and improves the quality of significant pathway set from original TRAP.

### Biological perspective

Table 3 shows part of pathways whose ranks went up as a result of incorporating the contextual information, compared to the original version of TRAP. An additional file shows the whole list of pathway rankings [see Additional file 3].

**Table 3:** The list of pathways that rank higher in ContextTRAP than in TRAP

Dataset	Pathway name	Rank	Description	Ref
*H5N1*	JAK-STAT signaling pathway	3→2	The challenging respiratory epithelial cells with hemagglutinin of H5N1 exploit the JAK2/3/STAT1 and result in a large release of cytokines, initiating a destructive innate immune response.	[36]
	PI3k-Akt signaling pathway	6→5	PI3K-Akt signaling, which can be activated by the NS1 protein of H5N1, is crucial for viral replication.	[43]
	Apoptosis	139→31	Apoptosis plays a major role in the pathogenesis of H5N1 virus in humans by destroying alveolar epithelial cells.	[44]
*Th17*	Cytokine-cytokine receptor interaction	33→10	The differentiation of *T*_*H*_17 cells from naive CD4 ^+^ T cells is regulated by multiple cytokines.	[38]
	MAPK signaling pathway	47→40	MAPKs play a supplemental role in mediating the intracellular responses to TGF- *β* required for differentiation of *T*_*H*_17.	[39]
	Toll-like receptor signaling pathway	46→41	Differentiation of *T*_*H*_17 cell is induced by proinflammatory cytokines generated by ligation of a subset of toll-like receptors.	[41]

It represents part of the pathways from *H5N1* and *Th17* that rank higher in ContextTRAP than in original TRAP. Rank column shows how the rank of the pathway in original TRAP is changed in ContextTRAP. Relation between those pathways and the context of the dataset is described with reference

In *H5N1*, the rank of the pathways that are related to the immune system and affected by infection of H5N1 influenza moved up significantly. Invading to host, H5N1 viruses activate PI3K-Akt signaling pathway to increase their replication efficiency [32]. The NS1 protein of H5N1 virus binds to p85 *β*, a regulatory subunit consisting in PI3K, and induces activation of Akt [33]. It leads to promoting cell growth, cell cycle or other cellular processes that support replication of H5N1 [34]. In addition, nucleoprotein in H5N1 induces apoptosis in host cells for their efficiency of replication, by interacting with clusterin, antiapoptotic protein of host [35]. The hemagglutinin of H5N1 activates JAK-STAT signaling pathway associated with transcriptional activation of chemokines/cytokines genes and incurs a destructive innate immune response [36].

In *Th17*, pathways related to cytokines that induce differentiation of *T*_*H*_17 cell moved upwards. Differentiation of *T*_*H*_17 cell is induced by IL-6 and TGF- *β*1 together [37]. In addition, IL-23, IL-1 *β* and IL-21 play a role in amplifying the differentiation IL-6 and TGF- *β*1 [38]. Mitogen-activated protein kinases (MAPK) including ERK, JNK and p38 are related to mediation of the intracellular responses to TGF- *β* [39, 40]. Toll-like receptor (TLR) is also involved in differentiation of *T*_*H*_17 cell [41]. TLR2 has been implicated in promoting *T*_*H*_17 cell differentiation and proliferation [42].

## Conclusions

Many gene expression analysis methods are available for identifying significant pathways from transcriptome data. However, these methods are often misled by many genes that are involved in a number of pathways. To address the challenge, we developed a new computational framework to combine analysis of gene expression data and the literature information based on the contextual information provided by the user as keyword. We defined two scores, *Impact Score* and *Context Score*, to measure *significance* from the result of pathway analysis and to specify *relevance* from the result of literature search, respectively.

Our novel framework was implemented as ContextTRAP by utilizing two existing tools, TRAP and BEST. We evaluated the performance of ContextTRAP with two data sets, *H5N1* and *Th17*, in comparison with the state of the art pathway analysis tools in each of the three categories of pathway analysis tools: Fisher’s exact test representing ORA methods, GSEA [17] representing FCS methods, and CliPPER [18] representing PT-based methods. In terms of F_1_ scores, ContextTRAP achieved better performances than existing methods and than the original TRAP. One notable feature is that ContextTRAP automatically combines *Impact Score* and *Context Score* into a single score by setting *Discovery rate* dynamically. In extensive experiments with various weights, *Discovery rate* showed the maximum performance, which demonstrates the ability of ContextTRAP to combine analysis of transcriptome data and the literature information dynamically, depending on the level of literature knowledge related to the experiment.

We believe that ContextTRAP will be a very useful resource for the pathway based analysis of gene expression data for the time-series, since the user can specify the context of the biological experiment in a set of keywords.

## Additional files

Additional file 1 Criteria of gene selection and selected gene set. It provides how to select the gene sets to find relevant pathways from each dataset and the result of selection mentioned in section *Data processing*. (XLS 49 kb)

Additional file 2 Pathway list selected as relevant pathways. It provides list of pathways relevant to each dataset which are selected by DAVID using validated gene sets mentioned in section *Data processing* and given in Additional file 1. (XLS 29 kb)

Additional file 3 Ranked list of pathways resulted from TRAP and ContextTRAP. It provided ranking information of whole pathways in TRAP and ContextTRAP. It is mentioned in section *Biological perspective* with part of pathways whose ranks went up in ContextTRAP. (XLS 71 kb)

## References

[1] Jin L, Zuo XY, Su WY, Zhao XL, Yuan MQ, Han LZ, Zhao X, Chen YD, Rao SQ (2014). Pathway-based analysis tools for complex diseases: a review. Genomics Proteomics Bioinforma.

[2] Luo L, Peng G, Zhu Y, Dong H, Amos CI, Xiong M (2010). Genome-wide gene and pathway analysis. Eur J Hum Genet.

[3] Chen H, Tseng Y, Wang S, Tsai Y, Chang C, Kuo T, Yao W, Shieh C, Wu C, Kuo P (2015). The metabolome profiling and pathway analysis in metabolic healthy and abnormal obesity. Int J Obes.

[4] Tu Z, Wang L, Arbeitman MN, Chen T, Sun F (2006). An integrative approach for causal gene identification and gene regulatory pathway inference. Bioinformatics.

[5] Mieczkowski J, Swiatek-Machado K, Kaminska B (2012). Identification of pathway deregulation–gene expression based analysis of consistent signal transduction. PLoS ONE.

[6] Creixell P, Reimand J, Haider S, Wu G, Shibata T, Vazquez M, Mustonen V, Gonzalez-Perez A, Pearson J, Sander C (2015). Pathway and network analysis of cancer genomes. Nat Methods.

[7] Gambardella G, Moretti MN, de Cegli R, Cardone L, Peron A, di Bernardo D (2013). Differential network analysis for the identification of condition-specific pathway activity and regulation. Bioinformatics.

[8] Lim S, Park Y, Hur B, Kim M, Han W, Kim S (2016). Protein interaction network (pin)-based breast cancer subsystem identification and activation measurement for prognostic modeling. Methods.

[9] Zhang F, Guo X, Wu S, Han J, Liu Y, Shen H, Deng HW (2012). Genome-wide pathway association studies of multiple correlated quantitative phenotypes using principle component analyses. PLoS ONE.

[10] Wang X, Pyne S, Dinu I (2014). Gene set enrichment analysis for multiple continuous phenotypes. BMC Bioinforma.

[11] Chowdhury S, Sarkar RR (2015). Comparison of human cell signaling pathway databases–evolution, drawbacks and challenges. Database.

[12] Kanehisa M, Goto S (2000). Kegg: kyoto encyclopedia of genes and genomes. Nucleic Acids Res.

[13] Joshi-Tope G, Gillespie M, Vastrik I, D’Eustachio P, Schmidt E, de Bono B, Jassal B, Gopinath G, Wu G, Matthews L (2005). Reactome: a knowledgebase of biological pathways. Nucleic Acids Res.

[14] Schaefer CF, Anthony K, Krupa S, Buchoff J, Day M, Hannay T, Buetow KH (2009). Pid: the pathway interaction database. Nucleic Acids Res.

[15] Khatri P, Sirota M, Butte AJ (2012). Ten years of pathway analysis: current approaches and outstanding challenges. PLoS Comput Biol.

[16] Rivals I, Personnaz L, Taing L, Potier MC (2007). Enrichment or depletion of a go category within a class of genes: which test?. Bioinformatics.

[17] Subramanian A, Tamayo P, Mootha VK, Mukherjee S, Ebert BL, Gillette MA, Paulovich A, Pomeroy SL, Golub TR, Lander ES (2005). Gene set enrichment analysis: a knowledge-based approach for interpreting genome-wide expression profiles. Proc Natl Acad Sci U S A.

[18] Martini P, Sales G, Massa MS, Chiogna M, Romualdi C (2013). Along signal paths: an empirical gene set approach exploiting pathway topology. Nucleic Acids Res.

[19] Bar-Joseph Z, Gitter A, Simon I (2012). Studying and modelling dynamic biological processes using time-series gene expression data. Nat Rev Genet.

[20] Jo K, Kwon HB, Kim S (2014). Time-series rna-seq analysis package (trap) and its application to the analysis of rice, oryza sativa l. ssp. japonica, upon drought stress. Methods.

[21] Tarca AL, Bhatti G, Romero R (2013). A comparison of gene set analysis methods in terms of sensitivity, prioritization and specificity. PLoS ONE.

[22] Jiang Z, Gentleman R (2007). Extensions to gene set enrichment. Bioinformatics.

[23] Donato M, Xu Z, Tomoiaga A, Granneman JG, MacKenzie RG, Bao R, Than NG, Westfall PH, Romero R, Draghici S (2013). Analysis and correction of crosstalk effects in pathway analysis. Genome Res.

[24] Holmans P (2009). Statistical methods for pathway analysis of genome-wide data for association with complex genetic traits. Adv Genet.

[25] Brodie A, Tovia-Brodie O, Ofran Y (2014). Large scale analysis of phenotype-pathway relationships based on gwas results. PLoS ONE.

[26] Lee S, Kim D, Lee K, Choi J, Kim S, Jeon M (2016). BEST: next-generation biomedical entity search tool for knowledge discovery from biomedical literature. PLoS ONE.

[27] Huang DW, Sherman BT, Lempicki RA (2008). Systematic and integrative analysis of large gene lists using david bioinformatics resources. Nat Protoc.

[28] Huang DW, Sherman BT, Lempicki RA (2009). Bioinformatics enrichment tools: paths toward the comprehensive functional analysis of large gene lists. Nucleic Acids Res.

[29] Li C, Bankhead A, Eisfeld AJ, Hatta Y, Jeng S, Chang JH, Aicher LD, Proll S, Ellis AL, Law GL (2011). Host regulatory network response to infection with highly pathogenic h5n1 avian influenza virus. J Virol.

[30] Yosef N, Shalek AK, Gaublomme JT, Jin H, Lee Y, Awasthi A, Wu C, Karwacz K, Xiao S, Jorgolli M (2013). Dynamic regulatory network controlling th17 cell differentiation. Nature.

[31] Sales G, Calura E, Martini P, Romualdi C (2013). Graphite web: Web tool for gene set analysis exploiting pathway topology. Nucleic Acids Res.

[32] Zhang D-g, Li W-z, Wang G-f, Su Y, Zeng J, Zhang C, Zeng X-x, Chen X-x, Xu Y-x, Li K-s (2010). Heterologous sh3-p85b inhibits influenza a virus replication. Virol J.

[33] Hale BG, Jackson D, Chen YH, Lamb RA, Randall RE (2006). Influenza a virus ns1 protein binds p85 *β* and activates phosphatidylinositol-3-kinase signaling. Proc Natl Acad Sci.

[34] Dunn EF, Connor JH (2012). Hijakt: The pi3k/akt pathway in virus replication and pathogenesis. Prog Mol Biol Transl Sci.

[35] Tripathi S, Batra J, Cao W, Sharma K, Patel J, Ranjan P, Kumar A, Katz J, Cox N, Lal R (2013). Influenza a virus nucleoprotein induces apoptosis in human airway epithelial cells: implications of a novel interaction between nucleoprotein and host protein clusterin. Cell Death Dis.

[36] Xu W, Chen M, Ge N, Xu J (2012). Hemagglutinin from the h5n1 virus activates janus kinase 3 to dysregulate innate immunity. PLoS ONE.

[37] Bettelli E, Carrier Y, Gao W, Korn T, Strom TB, Oukka M, Weiner HL, Kuchroo VK (2006). Reciprocal developmental pathways for the generation of pathogenic effector th17 and regulatory t cells. Nature.

[38] Zheng X, Bian F, Ma P, De Paiva CS, Stern M, Pflugfelder SC, Li DQ (2010). Induction of th17 differentiation by corneal epithelial-derived cytokines. J Cell Physiol.

[39] Lu L, Wang J, Zhang F, Chai Y, Brand D, Wang X, Horwitz DA, Shi W, Zheng SG (2010). Role of smad and non-smad signals in the development of th17 and regulatory t cells. J Immunol.

[40] Di Mitri D, Sambucci M, Loiarro M, De Bardi M, Volpe E, Cencioni MT, Gasperini C, Centonze D, Sette C, Akbar AN (2015). The p38 mitogen-activated protein kinase cascade modulates t helper type 17 differentiation and functionality in multiple sclerosis. Immunology.

[41] Kattah MG, Wong MT, Yocum MD, Utz PJ (2008). Cytokines secreted in response to toll-like receptor ligand stimulation modulate differentiation of human th17 cells. Arthritis Rheum.

[42] Bird L (2010). T cells: Tlrs deliver a direct hit to th17 cells. Nat Rev Immunol.

[43] Wei K, Chen Y, Lin Y, Pan Y (2014). Genetic dynamic analysis of the influenza a h5n1 ns1 gene in china. PLoS ONE.

[44] Uiprasertkul M, Kitphati R, Puthavathana P, Kriwong R, Kongchanagul A, Ungchusak K, Angkasekwinai S, Chokephaibulkit K, Srisook K, Vanprapar N (2007). Apoptosis and pathogenesis of avian influenza a (h5n1) virus in humans. Emerg Infect Dis.

